# Environmental Modeling and Exposure Assessment of Sediment-Associated Pyrethroids in an Agricultural Watershed

**DOI:** 10.1371/journal.pone.0015794

**Published:** 2011-01-05

**Authors:** Yuzhou Luo, Minghua Zhang

**Affiliations:** 1 Wenzhou Medical College, Wenzhou, China; 2 Department of Land, Air, and Water Resources, University of California Davis, Davis, California, United States of America; University of Kansas, United States of America

## Abstract

Synthetic pyrethroid insecticides have generated public concerns due to their increasing use and potential effects on aquatic ecosystems. A modeling system was developed in this study for simulating the transport processes and associated sediment toxicity of pyrethroids at coupled field/watershed scales. The model was tested in the Orestimba Creek watershed, an agriculturally intensive area in California' Central Valley. Model predictions were satisfactory when compared with measured suspended solid concentration (R^2^ = 0.536), pyrethroid toxic unit (0.576), and cumulative mortality of *Hyalella azteca* (0.570). The results indicated that sediment toxicity in the study area was strongly related to the concentration of pyrethroids in bed sediment. Bifenthrin was identified as the dominant contributor to the sediment toxicity in recent years, accounting for 50–85% of predicted toxicity units. In addition, more than 90% of the variation on the annual maximum toxic unit of pyrethroids was attributed to precipitation and prior application of bifenthrin in the late irrigation season. As one of the first studies simulating the dynamics and spatial variability of pyrethroids in fields and instreams, the modeling results provided useful information on new policies to be considered with respect to pyrethroid regulation. This study suggested two potential measures to efficiently reduce sediment toxicity by pyrethroids in the study area: [1] limiting bifenthrin use immediately before rainfall season; and [2] implementing conservation practices to retain soil on cropland.

## Introduction

Use of pesticides in crop production has been an important practice in modern agriculture, especially in the Central Valley of California, the most dynamic agricultural region in the world. Pesticide use can lead to severe environmental problems due to their toxicity to humans and many ecosystem organisms. Synthetic pyrethroids have become increasingly popular following outright bans or limitations on the use of cholinesterase-inhibiting insecticides, such as organophosphates (OPs). Previous studies have indicated that the decrease in OP use in California was related to the substitution with pyrethroids [1]. Pyrethroid insecticides are associated with selective potency in insects and relatively low potency in mammals. However, results of exposure monitoring and pesticide illness surveillance suggested that field residues of pyrethroids can cause irritant respiratory symptoms, nausea and headache [2]. Furthermore, pyrethroids are very acutely toxic to fish and invertebrates, with the 10-day LC50 values ranging from 2–140 ng/L in water (*Americamysis bahia* and *Ceriodaphnia dubia*) and 4–110 ng/g in sediment (*Hyalella azteca*) [3]. Surface water monitoring indicated widespread presence of pyrethroids and associated toxicity in agricultural and urban waterways in California [4], [5], [6]. Identifying the distribution of pyrethroids in surface waters and their effects on aquatic organisms is very important in pesticide regulation and water management of pyrethroids.

Monitoring data are usually insufficient to characterize the spatial distribution and the main sources of pesticide residues. Therefore, mathematical models are used to simulate the effects of pesticide use, management practices, and environmental factors on pesticide fate and distribution. In addition, the regulatory burden has evolved to currently consider negative impacts of pesticides on aquatic organisms. Detailed information on pesticide residues, such as the magnitude, timing and frequency of peak concentrations, are required to examine the overall ecosystem exposure by the use of pesticides. Therefore, continuous modeling at the field scale is essential for decision making processes to adequately meet regulatory requirements and improve management practices.

Recent developments in GIS technology enable the application of field-scale models on a large landscape by incorporating spatially distributed simulations of water and chemical movement in river networks. The integrated systems with field-scale models routing algorithms have been successfully applied to simulate pesticide fate and behaviors in streams. Most of those models were originally designed for the simulation of pesticides in the dissolved phase, indicating appropriate model applications on pesticides with lower adsorption coefficients. With octanol-water partition coefficients (K_OW_) values of 10^5^–10^7^, pyrethroids tend to adsorb to soil and sediment rather than remain in the dissolved phase [7]. Therefore, accurate prediction of pyrethroid fate and transport must incorporate the simulations of soil erosion, in-stream sediment transport, and pyrethroid partitioning. Due to inadequate representation of the above hydrologic and transport processes, most of the existing field-scale models and in-stream routing models are not appropriate for predicting environmental behaviors of pyrethroids. For example, RZWQM (Root zone water quality model) were developed for water flow and solute transport, and thus do not simulate soil erosion and adsorbed pesticide removal. Consequently, edge-of-field pesticide fluxes are underestimated, especially for those with strong sediment/soil sorption [8]. In the GLEAMS (Groundwater loading effects of agricultural management system) model, solid-bound pesticide concentration in eroded soil is determined based on a prescribed soil mass per unit runoff volume regardless of the actual soil erosion rate [9]. In addition, many popular routing models are not able to sufficiently capture the dynamics of pesticide partitioning and transport. They either assume a steady state hydraulics (e.g., River and stream water quality model, QUAL2K [10]), or utilize prescribed suspended solid concentrations (e.g., River water quality model, RIVWQ [11]).

**Figure 1:**
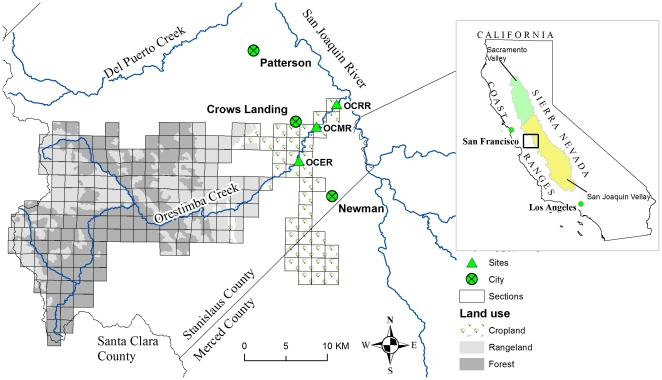
Orestimba Creek watershed and the sampling sites.

PRZM (Pesticide root zone model) estimates soil erosion based on a modified Universal Soil Loss Equation. In our previous study [12], PRZM model was coupled with a linear routing model for assessing pesticide dynamics and distribution in crop fields and stream networks. The coupled system provided a suitable modeling platform for determining environmental concentration and toxicity of pyrethroids. However, some model improvements were required. For example, a minor deficiency has been identified in the PRZM algorithm for adsorbed pesticide removal [13]. This paper presents an improved modeling system based on our previous study [12] for simulating the environmental fate and dispersion of pyrethroid insecticides. The specific purposes for the proposed model were to: (a) account for pyrethroid entry into surface water via soil erosion, (b) predict dynamics and distribution of pyrethroids in channel flow and bed sediment, and (c) characterize the toxicity by pyrethroids to sediment-dwelling organisms. This is one of the first studies on the dynamic modeling of pyrethroids at watershed scale, responding to the emerging research need for pyrethroid reevaluation and watershed management planning. Simulation capability of the developed model was demonstrated by applying it to the Orestimba Creek Watershed (Figure 1), an agriculturally dominated watershed in the California's Central Valley, with four pyrethroids of bifenthrin, λ -cyhalothrin, esfenvalerate, and permethrin as test agents.

## Results and Discussion

### Evaluation of the improved algorithm

**Figure 2:**
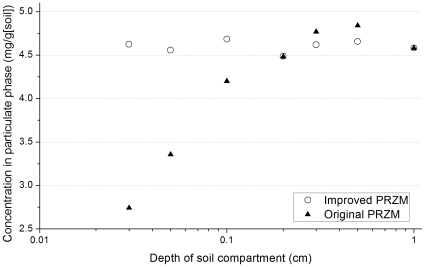
PRZM-simulated removals of absorbed pesticide with depths of soil compartment, tested with historical bifenthrin applications at a melon crop field in the study area.

The improved algorithm for adsorbed pesticide removal in PRZM was evaluated in a melon crop field with historical bifenthrin applications in the Orestimba Creek watershed. This field is close to the monitoring site OCER (Orestimba Creek at Eastin Road, Figure 1 and Table 1), with an average annual bifenthrin use of 0.08 kg per treated hectare. The concentration of eroded bifenthrin was reported as the total amount of eroded bifenthrin divided by the total amount of eroded soil during the simulation period. Results of the improved PRZM in this study were generally invariant with the depth of soil compartment used in the numerical analysis (Figure 2). This confirmed that the improved algorithm removed adsorbed pesticide from all compartments within the soil-interaction depth (D_E_, see Materials and Methods), thus the resulting removal was not dependent with the depth of each compartment. As discussed in Materials and Methods, the original PRZM considers only the top-most soil compartment for adsorbed pesticide removal; therefore, the results were very sensitive to the depth. The original PRZM generated similar results as the improved one when the depth of soil compartment was close to D_E_ (1 cm in this case study). Figure 2 demostrated PRZM simulations for soil erosion and associated pesticide removal with depth of soil compartment up to 1 cm. In the real PRZM modeling, however, small depth was required for accurate numerical simulation of water and chemical movement in the soil. For example, all crop scenarios for PRZM require depth of 0.1 cm or less for the top soil horizon developed by U.S. Environmental Protection Agency (USEPA) [14]. With small depth of soil compartment, the original PRZM significantly underestimated the adsorbed pesticide removal. The improved PRZM should be applied for consistence estimations of adsorbed pesticide release from the applied field.

**Table 1:** Sampling sites for model evaluation.

Name	Site ID	USGS ID	Latitude	Longitude
Orestimba Creek at Eastin Road	OCER	-	37.35	−121.07
Orestimba Creek at Morris Road	OCMR	-	37.39	−121.04
Orestimba Creek at River Road	OCRR	11274538	37.41	−121.02

### Sediment Loadings

**Figure 3:**
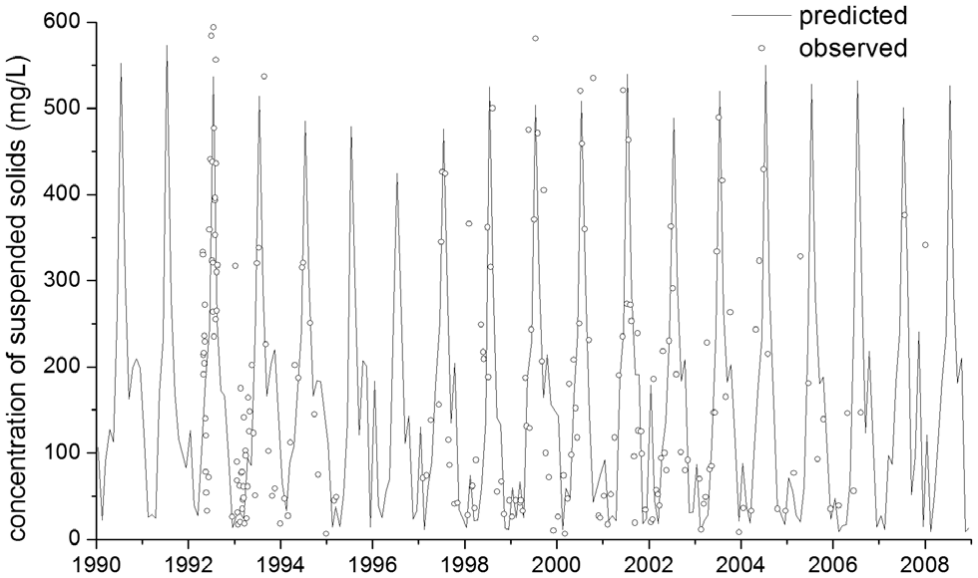
Observed and predicted monthly flow-weighted concentrations of suspended solids at the site OCRR (Orestimba Creek at River Road).

Due to their high adsorption coefficients (K_OW_), pyrethroids are typically adsorbed to soil particles and transported with suspended solids in surface runoff and stream flows. Therefore, a reasonable estimation of sediment concentration in a stream is the first necessary step in simulating pyrethroid partitioning between dissolved and particulate phases. Figure 3 shows the flow-weighted suspended solid concentration on a monthly basis observed and predicted at site OCRR (Orestimba Creek at River Road, close to the watershed outlet, Figure 1 and Table 1). The temporal trend of predictions followed the measured data (R^2^ = 0.536), indicating a satisfactory simulation of suspended solid transport processes based on the model evaluation guidelines by Moriasi *et al.* [15]. High concentrations of suspended solids were observed during the irrigation season, especially in July.

According to the USGS sampling results, in-stream concentrations of particulate organic carbon (OC) and suspended solids were strongly correlated (r = 0.90, p<0.001). Therefore, agreement between the predicted and observed concentrations of suspended solids also indicated a reasonable simulation for the particulate OC concentrations in the study area.

### Pyrethroid Toxicity

**Figure 4:**
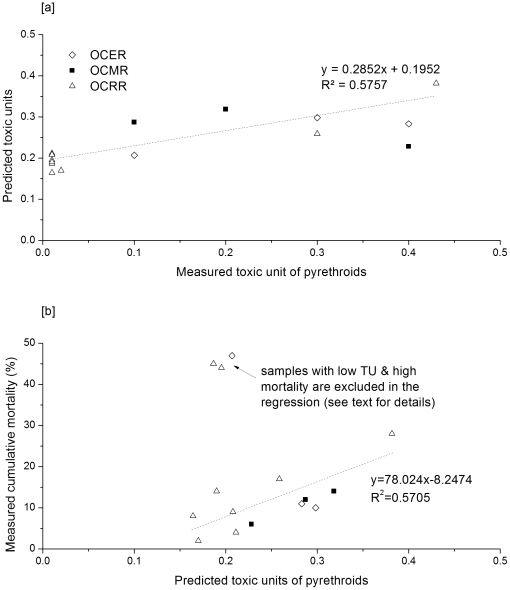
Predicted toxic units (TU) of the simulated pyrethroids in this study, in comparisons with [a] measured TUs (samples without detected pyrethroids are arbitrarily plotted on the figure at 0.01 TU), and [b] measured cumulative mortality.

Significant correlation (r = 0.72, p = 0.004) was observed between the predicted and measured toxicity units (TU) of pyrethroids in 15 samples at the three monitoring sites during 2007 and 2008 (Figure 4a). This indicated that the model generally captured the spatial variability and seasonality of the pyrethroid distribution in bed sediment. Based on the model prediction and field measurements, the OCRR site was generally associated with low pyrethroid TU relative to the other sites. Samples with undetected pyrethroids (plotted at 0.01 for measured TU in the figure) were all collected at the OCRR site during dormant seasons or early irrigation seasons. Located at the outlet of the Orestimba Creek, OCRR has larger drainage areas and a longer transport path for pesticides compared to other two sites. Pesticide residues have been largely decayed and diluted before reaching the water-sediment system at this site. For those undetected samples in the OCRR site, corresponding model results yielded TU values of 0.16–0.21, suggesting that the actual toxicity level was undetectable by the analytical methods applied in the sampling projects. This might also be the reason why the model overestimated measured data with low TUs. In the range of higher toxic levels (TU> = 0.3), the model had better agreement with the measurements, and the predicted and measured TUs approached the 1∶1 line on the plot.

Figure 4b compares the predicted TUs based on the simulated pyrethroids to the measured cumulative mortality of *Hyalella azteca*. For mortality values <40%, the predicted TUs are significantly correlated to the measured cumulative mortality (r = 0.75, p = 0.005). By fitting a toxicity-mortality curve in logistic form [16], the resultant R^2^ was 0.578, suggesting the model satisfactorily captured the dose-expose relationship in the evaluated sampling site. This also supported the hypothesis that sediment toxicity in the study area was mainly associated with pyrethroid concentrations. However, the correlation was not as strong among the samples with higher mortality values of >40%. For those samples, predicted TUs were about 0.2 based on the modeled pyrethroid concentrations in this study. This deficiency may have arisen due to the substantial contributions of other pesticides or other toxic compounds which were not modeled in this study to the measured sediment toxicity. This possibility was confirmed by the fact that, for those samples with high mortality, the measured TUs in sediment were also low, ranging from non-detected to 0.1. Another issue was that some samples with relatively high measured TUs were associated with low mortality. For instance, the sample at site OCMR in September 2007 had a measured TU of 0.4 and mortality of 6%. In this case, the respective model prediction of TU = 0.23 gave a more reasonable match to the observed mortality.

### Characterization of Pyrethroid Exposures

**Figure 5:**
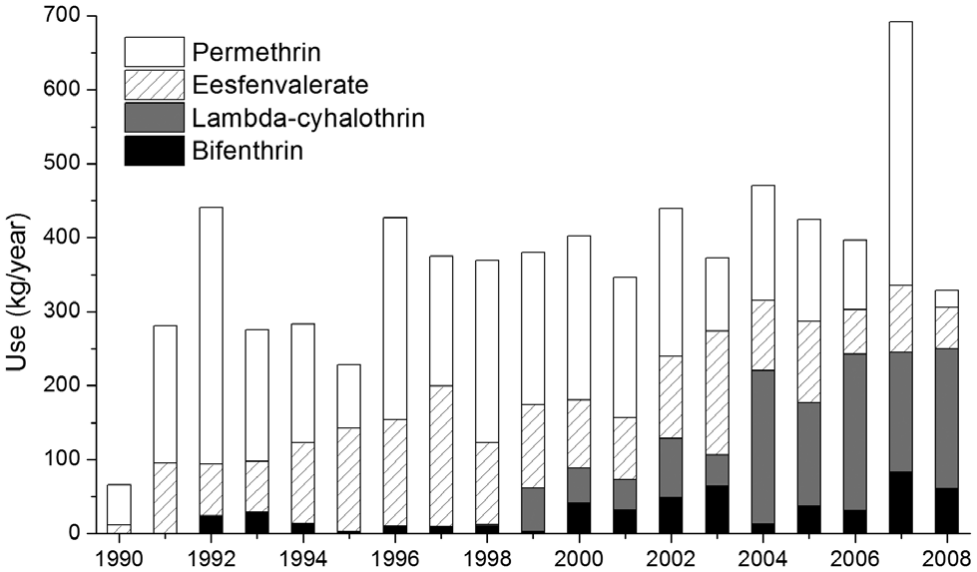
Annual uses of the simulated pyrethroids in the study area.

**Figure 6:**
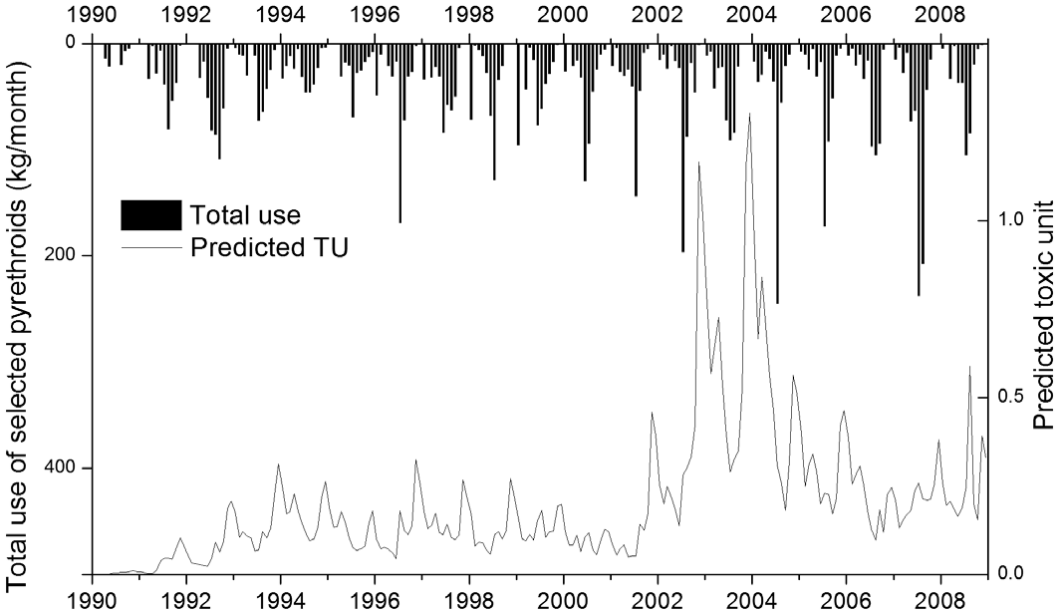
Predicted monthly toxic units (TU) of pyrethroids in bed sediment at the site OCRR (Orestimba Creek at River Road) and corresponding monthly pyrethroid uses in the drainage area.

In the Orestimba Creek watershed, there was a general increasing trend of total pyrethroid use during 1990–2004 (Figure 5). This increase was mainly attributed to esfenvalerate for years before 2000, and to bifenthrin and λ-cyhalothrin after 2000. After 2004, the amount of pyrethroids used has decreased, except for 2007 when reported permethrin use was very high. Figure 6 shows the predicted TUs at the OCRR site on a monthly basis, presenting sub-chronic risks of benthic organisms to pyrethroid exposures. Similar temporal trends of the predicted pyrethroid TUs were shared at the three sites in this study. Before 2000, the predicted sediment toxicity in the study was low, with maximum monthly TUs less than 0.5. Esfenvalerate was the major contributor to TUs during this period. The use of bifenthrin was started from July 1992 and explained a significant portion of the predicted TU. However, there was a general decreasing trend for both bifenthrin use (from 23.4 to 3.2 kg/year) and predicted TUs during 1992–1999. After 2000, bifenthrin use was increased again and predicted TU in sediment was substantially elevated and peaked in 2002–2004. Another potential reason for the elevated TUs was the change in application timing of bifenthrin. While it was mainly applied in July and August before 2000, significant amounts of bifenthrin were also applied during the late irrigation months of September and October and subject to the significant runoff events induced by winter precipitation. Consequently, bifenthrin became the major contributor to sediment toxicity for the last 10 years, accounting for 50–85% TU in sediment. Esfenvalerate and λ-cyhalothrin were also important contributors, especially during the irrigation season when they explained up to 50% of TU in sediment. During the recent years of 2004–2008, λ-cyhalothrin accounted for 38% of total pyrethroid used in the study area, followed by permethrin (32%), esfenvalerate (17%), and bifenthrin (10%). It is noteworthy that the use of λ-cyhalothrin was first reported in this area in 1998 and its use has been significantly increased since 2004, with an annual rate of about 180 kg in recent years. However, λ-cyhalothrin has a relatively short half-life in sediment (12 days), limiting its persistence and toxicity in aquatic ecosystems.

During the study period, about 50% of annual pyrethroids are applied in July and August, and 70% during June to September. However, high concentrations and TU values of pyrethroids in sediment were predicted during rainfall seasons. Predicted TUs from November to January were significantly higher than the annual average. Therefore, no linear relationship was confirmed for pyrethroid uses and predicted TUs on either monthly or annual bases. In previous studies, however, such correlations were reported for organophosphate pesticides [12], [17]. With relatively high adsorption coefficients, the off-site movement of pyrethroids is mainly associated with soil erosion from agricultural fields and suspended solid transport in channels. Bifenthrin is highly persistent in soil and sediment with half-lives in aerobic soils of 85 days and in aquatic sediments of 251 days used in the modeling (Table 2). The applied pyrethroids might persist in soil and sediment long enough, and be available for the subsequent winter storms. Therefore, it is possible that predicted TUs in sediment reflected pyrethroid uses in the previous growing season. Significant time-lagged correlations were detected between the annual maximum TU and total prior bifenthrin use during the late irrigation season of September and October (r = 0.74, p = 0.022) for years when bifenthrin usage in September and October were observed (Table 3). Monthly precipitation corresponding to the maximum TU was identified as the second most important factor. Further analysis indicated that the precipitation and September+October bifenthrin use explained 90.2% of the variance of the annual maximum TUs, among which 54.9% was contributed by the bifenthrin use and 35.3% by the precipitation. This finding suggested two potential measures to efficiently reduce sediment toxicity by pyrethroids in the study area: [1] limiting bifenthrin use immediately before rainfall season; and [2] implementing conservation practices to retain soil on cropland, which would mitigate suspended soild transport to surface water bodies during early rainfall season.

**Table 2:** Chemical and toxic data for the simulated pyrethroids.

Parameter (unit)	Bifenthrin	λ-cyhalothrin	Esfenvalerate	Permethrin
Molecular weight (g/mol)	422.9	449.85	419.9	391.3
Octanol-water partition coefficient (K_OW_, L/kg)	2.00E+07	7.94E+06	1.74E+06	1.26E+06
Vapor pressure (Pa)	1.78E−05	2.00E−07	1.20E−09	2.00E−06
Henry's law constant (Pa*m^3^/mol)	7.74E−05	2.00E−02	4.90E−04	1.89E−01
Organic-carbon normalized partition coefficient (L/kg)	2.37E+05	1.57E+05	5300	1.00E+05
Half-life (day)				
in soil	85	25	44	42
in water	8	8	30	23
in sediment	251	12	71	40
Method detection limit (ng/g)				
in Domagalski et al. [29]	2.2	2.4	2.1	1.0
in Ensminger et al. [30]	0.5	2.0	1.0	2.0
10-day LC50 (ng/g)	5.2	4.5	15.4	108

Notes:

[1] LC50  =  median lethal concentration for *Hyalella azteca* in sediment containing 1% organic carbon.

[2] Data sources: physicochemical properties and reaction half-lives were retrieved from FOOTPRINT pesticide properties database [35]. Method detection limits were taken from the respective studies. LC50 values were compiled by Domagalski et al. [29] from the literature.

**Table 3:** Precipitation, bifenthrin use, and predicted max TUs at the site OCRR during 1990–2008.

Year	Max TU (rainfall season)	Precipitation (cm)		Bifenthrin use (kg)	
		Nov & Dec	annual	Sep & Oct	annual
1990	0.0117	1.72	17.87	0.00	0.00
1991	0.1045	2.4	23.05	0.00	0.00
1992	0.2072	2.52	22.9	0.00	24.05
1993	0.3128	0.89	19.16	0.00	29.41
1994	0.2629	0.71	6.37	0.00	13.66
1995	0.1798	3.73	29.54	1.34	2.77
1996	0.3257	12.12	41.76	0.00	10.42
1997	0.2682	12.3	26.03	0.00	9.65
1998	0.2715	3.96	47.44	0.00	10.24
1999	0.1976	2.24	28.2	0.00	3.15
2000	0.1286	0.65	19.34	8.14	41.10
2001	0.4581	9.2	26.03	0.00	31.93
2002	1.1647	11.44	24.53	13.34	48.73
2003	1.3027	7.23	16.9	18.73	64.19
2004	0.5627	8.49	25.67	1.34	13.52
2005	0.4622	3.92	25.67	6.38	36.93
2006	0.2462	2.8	19.01	9.60	31.01
2007	0.3816	3.47	12.91	4.02	83.24
2008	0.3923	3.26	12.95	1.76	61.13

## Materials and Methods

### PRZM application at watershed scale

A geo-referenced modeling system has been developed in our previous study [12] for tracking pesticide transport from its field application to the receiving waters. Pesticide discharges from the soil-canopy system were simulated by PRZM model. PRZM [18] is a one-dimensional dynamic model, primarily designed to predict the influence of climate, land/soil properties, and agricultural management on the physical and biochemical dynamics of pesticides in the environment. PRZM was selected based on its ability to simulate relevant governing processes of pesticide transport and its preferential use by the USEPA for pesticide-associated risk assessment [19]. Pre-calibrated PRZM parameters were recommended in the USEPA Standard Tier 2 scenarios for the major crops throughout the United States [14]. GIS technology was used to extend the PRZM capability for geo-referenced parameterization and application at a watershed scale.

Based on a linear routing model, edge-of-field fluxes of water and pesticides predicted by PRZM were routed through stream channels to a downstream location, e.g., a monitoring site. For water transport, stream flows at the routing destination were calculated as the summation of convolutions between PRZM-predicted runoff and corresponding watershed unit hydrograph in each simulation zone. The hydrologic response was presented by a flow-path redistribution function (*U*) based on the first passage time distribution [20], [21],
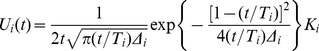
(1)where i is a running index for simulation zone, T (s) is the lag time in the flow-path, Δ_i_ (dimensionless) represents the shear and storage effects on the flow, and K_i_ (dimensionless) is the loss factor accounting for evaporation and transmission losses.

The same flow-path redistribution functions were also applied in the transport simulation of dissolved pesticides. A pesticide dispersion coefficient was determined as the sum of molecular diffusivity and flow diffusivity for the corresponding flow paths, and pesticide decay rate was calculated from its aquatic half-life. Modeling nodes in the channel network were selected to correspond with tributary/drainage junctions and monitoring locations. The developed model was applied to the Orestimba Creek watershed during 1990 through 2006, with diazinon and chlorpyrifos as test agents. The model yields reasonable agreements with measured data for the stream flow and dissolved pesticide loads [12].

### Pesticide transport with eroded soil

PRZM is known to inadequately predict pesticide transport associated with soil erosion [13]. In PRZM, soil column was divided into compartments according to user-defined numerical simulation interval of soil depth. Adsorbed pesticide is only removed from the top-most compartment. Therefore, the removal of pesticide in adsorbed phase is primarily a function of soil compartment depth. Small depths of soil compartments, which are likely to be applied by model users to improve the numerical calculations, will result in significantly less pesticide mass removed by erosion, especially for high-sorbing compounds such as pyrethroids.

In this study, we improved the PRZM simulation algorithm by introducing a soil-interaction depth (D_E_). It is assumed that all soil layers from the ground to the depth of D_E_ were subjected to the soil erosion process. This concept is similar to the extraction model used in transport model for estimating dissolved chemicals in surface runoff. For example, PRZM estimates the amount of dissolved pesticide runoff based on the average concentration of dissolved pesticide concentration weighted by an exponential curve for all compartments from the surface to a depth of 2 cm. The depth D_E_, which could be initialized and calibrated by users, is independent from the compartment size for numerical calculation, and remains a fixed value during each PRZM simulation run.

Weighted average concentration of pesticide adsorbed on soil particles subject to erosion (C_S,E_, g/g) for all compartments within the depth of D_E_ was first determined as:
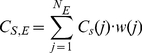
(2)where j is a running index for compartments, N_E_ is total compartments within D_E_, C_s_ (g/g) is the concentration of soil-bound pesticides, and w is a return-to-unit weighting function, i.e., sum(w)  = 1. The amount of adsorbed pesticide transported out of the field (J_ER_, g/day) is calculated by: 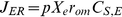
(3)


This equation was the same as Eq. (6.13) in the PRZM manual [18], with X_e_ (ton/day) as the erosion sediment loss, r_om_ as the enrichment ratio for organic matter, and p as a units conversion factor (g/ton). To implement the above equation, source codes of PRZM were modified and the new procedure for determining pesticide removal with eroded soil was:

[1] initialize the soil-interaction depth (D_E_), and define a weighting curve as a function of depth (w);
[2] determine the affected compartments within D_E_, and calculate weighting factors for each compartment;
[3] adjust “B” term in the PRZM numerical solution as:
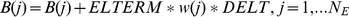
(4)where B (day^−1^) is the diagonal element in the tri-diagonal matrix solution (Thomas algorithm) utilized by the PRZM code for the governing equations of pesticide transport, ELTERM (day^−1^) is the erosion loss term for pesticide balance, and DELT (day) is the simulation time step.

### Sediment and pesticide transport in stream network

Chemical partitioning and degradation in channel transport have been formulated in the linear routing model as described in our previous study [12]. In this study, improvements were made mainly for sediment routing and partitioning/transport of pesticide associated with suspended solids and bed sediment. A concept of sediment transport capacity was applied in this study to predict sediment deposition in the channels. By following the algorithm of Soil and water assessment tool (SWAT) [22], the maximum sediment concentration (C_ss,max_, kg/m^3^) that can be transported from a reach segment is calculated as:
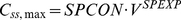
(5)where V (m/s) is the peak channel velocity, and SPCON and SPEXP are coefficients to be determined. Sedimentation flux was determined by comparing the initial concentration of suspended solids in a reach at a time step (C_ss,0_, kg/m^3^) to C_ss,max_. For instance, if C_ss,0_>C_ss,max_, the exceeding amount of suspended solids and associated pesticides in the adsorbed phase would be transported into bed sediment. The resulting sedimentation flux was used to adjust the initial concentration of suspended solids. A similar methodology was applied in the calculation of resuspension fluxes of bed sediment and pesticides by introducing factors for channel erodibility and channel cover [22]. The predicted concentrations of suspended solids were applied to determine pesticide partitioning between dissolved and adsorbed phases in the water column:
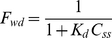
(6)


where F_wd_ (dimensionless) is the fraction of total pesticide of the water column in dissolved phase, K_d_ (L/kg) is the pesticide partition coefficient, and C_ss_ (kg/m^3^) is the predicted concentration of suspended solids.

Pesticide simulation in bed sediment was only conducted for the active sediment layer with user-defined depth. Based on the solid-liquid partitioning, the fraction (F_dd_, dimensionless) of total sediment pesticide in the dissolved phase was calculated as:
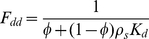
(7)


with Φ (dimensionless) denoting sediment porosity and ρ_s_ (g/m^3^) particle density. Pesticide decay and burial in bed sediment were combined and simulated according to first-order kinetics. Pesticide transport flux by sedimentation was calculated as the product of previously determined sedimentation flux of suspended solids and the pesticide concentration in suspended solids. Similarly, pesticide resuspension flux was based on the sediment resuspension flux and the concentration of sediment-bound pesticide. Therefore, sedimentation and resuspension processes for both suspended solids and solid-bound pesticides were simulated dynamically rather than being prescribed.

Pesticide diffusion flux (J_diff_, kg/m^2^/day) between the water and bed sediment was formulated using a multimedia environmental fate modeling approach [23]:
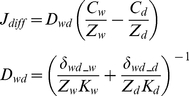
(8)where subscripts w and d are for water compartment and sediment compartment, respectively, D_wd_ (kg/Pa/day) is the Mackay-type mass transfer coefficient, the Z's (mol/Pa/m^3^) are the fugacity capacity of pesticide, δ's (m) are boundary layer depths at the water-sediment interface, and K's (m^2^/day) are pesticide diffusivities. As suggested by the CalTox model [24], the boundary layer thickness in water side (δ_wd_w_) was set as 0.02 m, while that in sediment side was estimated as 318K_d_^0.683^.

### Site Description

The modeling system newly developed in this study was applied to the field conditions of the Orestimba Creek watershed of California (Figure 1). Located in western Stanislaus County, the creek originates in the mountainous areas of the Coast Range, and discharges into the San Joaquin River. Characterized by heavier textured soils and greater slopes relative to eastside watersheds of the San Joaquin River, the Orestimba Creek watershed represents a worse-than-average condition for pesticide contamination in surface water. Climate and landscape characteristics for the studied watershed were summarized in the previous studies [12], [25].

The lower reach of the Orestimba Creek flows through agricultural lands in California's Central Valley, the most dynamic agricultural region in the world. Pyrethroids are applied to control a myriad of pests, and in this study the most important crops receiving these insecticides are orchards and row crops. Sediment from this creek was also found to be toxic to sediment-dwelling organisms, most likely because of high levels of pyrethroids [26]. Based on sediment sampling in the Orestimba Creek during 2004 irrigation season, high sediment concentrations of bifenthrin and λ-cyhalothrin were reported with acute toxicity to sensitive aquatic species [27]. In the 2010 Clean Water Act 303(d) report of California, the Orestimba Creek was listed for sediment toxicity, however the source pollutants were not yet fully identified in the report [28].

### Pesticide Data Acquisition

The case study was based on two monitoring studies of pyrethroid concentrations and aquatic toxicity in streambed sediments of the Orestimba Creek watershed. Our previous monitoring study included 20 sampling sites throughout the San Joaquin River Valley, with 3 sites situated within the Orestimba Creek watershed [29] (Figure 1 and Table 1). Field measurements were conducted for 9 pyrethroids during the irrigation season of 2007. In an associated study, Ensminger et al. [30] collected monthly water and sediment samples at the site OCRR from December 2007 through June 2008, to determine concentrations of organophosphate and pyrethroid insecticides. In addition to chemical analyses, both studies conducted sediment toxicity tests with *Hyalella azteca*, following the standard USEPA protocols [31]. More details on experimental design, analytical and sediment toxicity methods, and monitoring results for the two studies can be found in Domagalski et al. [29] and Ensminger et al. [30].

Among all analyzed pyrethroids, only those detected at least twice in the two sampling studies, including bifenthrin, λ -cyhalothrin, esfenvalerate, and permethrin, were selected for model application in this study. Table 2 lists the physicochemical properties, reaction half-lives, and toxicity benchmark (as 10-d median lethal concentration, LC50, for *Hyalella azteca* in sediment) of the simulated pyrethroids. Pesticide application data were retrieved from the Pesticide Use Reporting (PUR) database maintained by California Department of Pesticide Regulation [32]. The PUR database records daily pesticide use by active ingredient and crops for each Meridian-Township-Range-Section (MTRS, or section) following the United States Land Survey System.

### Simulation Design

The improved PRZM and routing simulations were performed to simulate water, sediment, and pesticide transport processes in the Orestimba Creek watershed at a daily time step for the period 1990–2008. The watershed was delineated into sections for the convenience of incorporating pesticide use data from the PUR database. Multiple fields were simulated in each section, based on the contemporary land use mapping in the study area [33]. A soil interaction depth of 1 cm and uniform weighting factors for each soil compartment were used in the case study, as suggested by SWAT documentation [22]. Individual conservation practices were not included in the model configuration. Instead, the model was calibrated based on the field measurements of water flow, sediment loading, and pesticide concentrations. Therefore, the model parameterization and simulation results reflected the overall reduction of pesticide use due to various best management practices (BMPs) implemented in the study area.

Channel parameters, including Manning's roughness coefficient, flow diffusivity, coefficients for sediment transport capacity, and depth for active sediment layers, were taken from previous studies in the Orestimba Creek watershed [17], [25]. Automatic calibration was conducted for the USLE crop factor (USLE_C) to match the measured suspended solid concentrations at the watershed outlet. The calibrated model was assumed to establish a reliable hydrologic framework for the study area, and applied to the dynamic simulation of pyrethroids.

### Model Evaluation

The modeling system has been validated in our previous study for its simulation capacity for stream flow and organophosphate pesticides in the dissolved phase [12]. In this study, therefore, model evaluation was emphasized on transport simulation of the suspended solids and absorbed pesticides. The location of the site OCRR is also gauged by a USGS station (#11274538) for stream flow, suspended solids, and organic carbon in suspended solids [34]. Flow-weighted concentrations of suspended solids and associated organic carbon on a monthly basis were calculated from the measurements at sampling days.

In the chemical analysis of pyrethroids, reported results were associated with different method detection limits (MDLs) (Table 2), and chemicals with concentrations lower than MDLs were reported as zeros. In addition, most of the reported concentrations of detectable pyrethroids in the study area were below the nominal reporting limit [29]. Therefore, it is not appropriate to directly compare predicted and observed concentrations for individual pyrethroids. In this study, predicted and observed pyrethroid concentrations were first converted into toxic units (TU), which is based on the assumption of toxicity additivity and is widely used as an estimate for aquatic toxicity. For each sample, the TU value was calculated as a summation of concentrations normalized by the corresponding sediment LC50 on an organic carbon (OC) basis. When no pyrethroids were detectable, the TU value was set as 0.01 for plotting convenience. To evaluate the model efficiency in predicting pyrethroid transport, the predicted TU values were compared with the measured values for each monitoring day at the three sites. It is important to note that the predicted TU values were calculated based on daily average predictions of pyrethroid concentrations, while measured values were from instantaneous samples. The model evaluation also compared the predicted TUs and cumulative mortality of *Hyalella azteca* to collected bed sediment samples. The cumulative mortality reflected the actual sediment toxicity by all chemicals, including those not analyzed or not detected, in the bed sediment. Thus, comparisons between predicted TUs and mortality were anticipated to provide useful information on the toxicity identification evaluations.
